# Risk Perception among Psychiatric Patients during the COVID-19 Pandemic

**DOI:** 10.3390/ijerph19052620

**Published:** 2022-02-24

**Authors:** Antimo Natale, Carmen Concerto, Alessandro Rodolico, Andrea Birgillito, Marina Bonelli, Miriam Martinez, Maria Salvina Signorelli, Antonino Petralia, Carmenrita Infortuna, Fortunato Battaglia, Eugenio Aguglia

**Affiliations:** 1 Psychiatry Unit, Department of Clinical and Experimental Medicine, University of Catania, 95123 Catania, Italy; antimo.natale@yahoo.it (A.N.); c.concerto@policlinico.unict.it (C.C.); alessandro.rodolico@phd.unict.it (A.R.); andrea.birgillito@gmail.com (A.B.); marina.bonelli@yahoo.it (M.B.); miriam.martinez@outlook.it (M.M.); maria.signorelli@unict.it (M.S.S.); petralia@unict.it (A.P.); eugenio.aguglia@unict.it (E.A.); 2 Department of Biomedical and Dental Sciences, Morphological and Functional Images, University of Messina, 98121 Messina, Italy; carmen.infortuna@gmail.com; 3 Department of Medical Sciences and Neurology, Hackensack Meridian School of Medicine, Nutley, NJ 07110, USA

**Keywords:** risk perception, COVID-19 pandemic, psychiatric patients

## Abstract

The fear of the new coronavirus infection has driven many non-COVID-19 patients away from essential healthcare. Our study aimed to investigate the perception of risk and feelings of danger for the contagion in a sample of Italian psychiatric patients. We conducted a cross-sectional observational study during the second wave of the COVID-19 pandemic and the lockdown in Italy. The risk perception and risk-related variables were assessed in a sample of 150 consecutive patients with a previous diagnosis of major depression (50), bipolar I disorder (50), and schizophrenia seeking ambulatory care using a questionnaire previously administered to the general Italian population. Our results showed that patients were more concerned about economic, psychological, and interpersonal consequences of COVID-19 pandemic, rather than about their own health. At the multiple regression analysis, the likelihood of COVID-19 resolution was positively influenced by the diagnosis of schizophrenia, the increased perceived effectiveness, and the presence of positive emotions. Indeed, positive emotions and uncertainty positively influenced interpersonal risk. Our study highlights the need to provide more support to psychiatric patients during emergency events to prevent them from engaging in risky behaviors.

## 1. Introduction

The coronavirus disease 2019 (COVID-19) infection was first diagnosed in China in December 2019. Subsequently, COVID-19 has spread throughout the world and in January 2020, the World Health Organization (WHO) declared a state of pandemic in Italy. Since then, in an attempt to contain the further spread of the virus, the Italian Government introduced strict measures like lockdowns, social distancing, limited access to several public places and activities, such as restaurants, theatres, cinemas, and sporting events [1]. The lack of social support due to the limited interaction with family and friends generated great psychological distress and forced people to seek new strategies to cope with the emergency situation and to adopt risk reduction behaviors [2,3].

The American Psychological Association (APA) defines the perception of risk as an “*individual’s subjective assessment of the level of risk associated with a particular hazard*” (e.g., health threat) [4]. The perception of risk has been previously investigated in different research fields [5,6,7] and the results indicate that it comprises both a rational, decision-making component, and a more instinctive reaction when facing danger [8,9]. Numerous health behavior theories indicate that the perception of risk is associated with the engagement of preventive behaviors [10,11,12]. Thus, the study of the measures of risk perception in the pandemic and the most common predictors of higher perceived risk of infections are of paramount importance, predicting future behavior in both the general population and specific patients’ groups. Several factors may influence the perception of risk in the general population including demographic variables (sex, ethnicity, and socioeconomic status [13,14]), social, and cultural processes [15,16]. Scholars have demonstrated that people would feel less vulnerable if they were better informed about the danger of an event [17,18]. On the other hand, excessive media exposure during a pandemic may enhance the feeling of danger and anxiety [19]. Furthermore, individual differences in the ability to adopt specific healthy behaviors to maintain good health during a risky situation [20,21] might affect the perception of risk during the COVID-19 pandemic [22,23].

The aforementioned studies indicate that research on risk perception of COVID-19 focused mostly on the general population, and little is known about the perception of risk during the COVID-19 pandemic in individuals affected by medical conditions. Stress and negative thoughts have been shown to have a detrimental effect on cortical plasticity and metaplasticity [24,25,26,27], which are impaired in patients with mental conditions and are shifted to physiologic levels by neuromodulatory treatments [28,29]. For instance, evidence suggests that patients with pre-existing mental disorders are more vulnerable to stressors [30]. Furthermore, the restrictions, with the consequent difficulty in reaching mental health services, add to the patient’ psychological burden [31]. Overall, data indicate that psychiatric patients are severely affected by the epidemic [32] and that the assessment of their concerns regarding the contagion are of pivotal importance for optimizing preventive patient ‘care.

In patients with a pre-existing psychiatric diagnosis of Major Depression (MDD), Bipolar I Disorder (BD), and schizophrenia (S) continuity of care is essential to ensure adherence to treatment and to prevent relapse. After a partial lifting of the restrictions during the second wave of the pandemic in Italy and the opening of outpatient appointments at the hospital, we sought to investigate whether patients with MDD, BD, and S were reluctant to visit the hospital due to fear of the infection. Recently, Lanciano et al. [33] developed an instrument to assess risk perception linked to the COVID-19 pandemic, taking into consideration several dimensions such as health, work, institutional-economy, interpersonal, and psychological. Hence, using this instrument, we sought to:

- investigate the perception of the likelihood of COVID-19 pandemic resolution in psychiatric patients seeking ambulatory care;
- determine differences in health risk concerns and health risk likelihood in patients with different pre-existing mental disorders;
- investigate the mortality risk perception, the economic risk perception, the interpersonal, and psychological risk perception;
- investigate the perceived knowledge regarding the pandemic and the perceived control concerning the risk for infection
- investigate their perception regarding the political decisions adopted to address the health crisis;
- investigate the patient ‘affective states during the COVID-19 pandemic.

We hypothesized that resuming ambulatory care during the second wave of the pandemic in Italy would be associated with substantial perception of the risk of COVID-19 infections and remarkable concerns regarding the patient’s financial and psychological burden.

## 2. Materials and Methods

### 2.1. Study Design

We included in the study 150 consecutive patients with a psychiatric diagnosis of Major Depression (50), Bipolar I Disorder (50), and Schizophrenia (50) requesting ambulatory care after the re-opening of the medical facilities in our institution during the second wave of the pandemic. The patients were previously tested through Drug Attitude Inventory (DAI), Quality of Life Index (QLF), Insight Scale (IS), and Short Form-12 Health Survey (SF-12). In addition, the severity of Schizophrenia was tested with the Positive and Negative Syndrome Scale (PANSS) and, in accordance with Andreasen criteria, a remission state was considered when the score was ≤3 in the 8 items (P1, G9, P3, P2, G5, N1, N4, N6) [34]. Severity of depression was evaluated with the Montgomery-Asberg Depression Rating Scale (MADRS), and remission was considered with values ≤10. Finally, bipolar patients were assessed with the MADRS and Young Mania Rating Scale (YMRS) patients with scores ≤10 for the MADRS and ≤7 for YMRS were considered clinically stable [35].

To assess the perception of risk, we used a previously validated risk assessment perception of COVID-19 in the general Italian population [33]. The questionnaire includes both items to assess demographic characteristics and risk perception:

- Socio-Demographics Characteristics: we collected the participant’s age, gender, marital status, education, employment, compliance with government regulations about quarantine, the presence of children at home, and the number of housemates during quarantine.
- Risk Perceptions:
  ⚬ *Likelihood of COVID-19 Resolution*: participants had to indicate the probability of the complete resolution of the pandemic and the likelihood of returning to previous life habits. It consists of two 11-point Likert items (0 = not at all; 10 = very much).
  ⚬ *Health Risk Perception Concern*: patients had to indicate how concerned they felt for their own health, for the health of their loved ones. They also had to express their concern to return to their old habits given the risk of infection. It consists of three 11-point Likert items (0 = not at all; 10 = very much).
  ⚬ *Health Risk Perception Likelihood*: we asked participants to provide an estimate of the likelihood of contagion, death, and recovery for themselves and others. It consists of 6 items and the participant answered by a 11-point scale (0 = not at all; 10 = very much);
  ⚬ *Work Risk Perception*: we asked participants to rate the impact of pandemic on unemployment, job management, job prospects, job self-efficacy, and employment relationships in the future. It is composed by five 11-point Likert items (0 = not at all; 10 = very much).
  ⚬ *Institutional-Economy Risk Perception:* the participants had to indicate a possible future role of the pandemic on continuity of government, EU relations, and political landscape or financial crisis. It is composed by four 11-point Likert items (0 = not at all; 10 = very much).
  ⚬ *Interpersonal Risk Perception*: we asked participants to estimate the effect of the pandemic on friendships, family relationships, love relationships, and social cohesion. It is composed by four 11-point Likert items (0 = not at all; 10 = very much).
  ⚬ *Psychological Risk Perception*: participants had to express how the pandemic will affect people’s freedom, self-actualization, well-being, isolation, and thinking modalities. It is composed by five 11-point Likert items (0 = not at all; 10 = very much).
  ⚬ *Mortality Risk Perception*: we asked participants about the likelihood of dying from the following cause: COVID-19, heart attack, stroke, cancer, dementia, and infection. It is composed by six 11-point Likert items (0 = not at all; 10 = very much).
  ⚬ *Perceived Knowledge*: we asked participants how well informed they felt regarding COVID-19. It is composed by one item and the participant answered by a 11-point Likert scale (0 = not at all; 10 = very much);
  ⚬ *COVID-19 Cause:* participants had to choose from a list of probable causes of COVID-19, the one that was most likely for them;
  ⚬ *News Seeking*: participants were asked how many times a day they searched for COVID-19 information (1 = never; 2 = 1 to 5 times; 3 = 5 to 10 times; 4 = more than 10 times);
  ⚬ *News Source*: participants indicated their news source of choice used to keep up to date (social networks, chat, institutional channels, newspapers, informal channels, websites, radio, etc.). Participant could choose multiple answers;
  ⚬ *Perceived Control*: participants had to express how much they could control the risk of infection. It is composed by one item and the participant answered by a 11-point Likert scale (0 = not at all; 10 = very much);
  ⚬ *Perceived Efficacy of Containment Measures*: we asked the participants to estimate the efficacy of government measures, the efficacy of compliance with government containment measures, perceived safety by respecting government containment measures, and efficacy of the contribution of each individual citizen during lockdown. It is composed of four items and the participant answered by a 11-point Likert scale (0 = not at all; 10 = very much);
  ⚬ *Affective States*: we asked the participants to rate each emotion they felt during the lockdown using an 11-point Likert scale (0 = not at all; 10 = very much) anger, wrath, fear, anguish, sadness, depression, loneliness, nostalgia (collected in a category “Negative Affective States index”); nervousness, anxiety, restlessness, vulnerability, (collected in a category “Anxiety States index”); impotence, frustration, inadequacy, uncertainty, confusion, disorientation (collected in a category “Uncertain States Index”); hope, and trust (collected in a category “Positive Affective States Index”).

The study was conducted according to the Declaration of Helsinki guidelines and approved by the University of Catania Psychiatry Unit review board (n. 1/2021)

### 2.2. Statistical Analysis

We checked for normality distribution of socio-demographic characteristics and all the continuous variables with Kolmogorov–Smirnov test. Data not normally distributed is reported as median and interquartile ranges, meanwhile categorical variables are presented as counts and percentages. We compared not normally distributed variables by diagnosis using Kruskal–Wallis tests. Chi-squared test was used to compare categorical variables. We created a correlation matrix comparing risk perception questionnaire items with each other, obtaining Spearman’s correlation coefficients, considering the whole sample and dividing participants by diagnosis separately. Multiple univariate regression models were run to identify if any risk perception variable score (likelihood of COVID-19 resolution, health risk perception concern, health risk perception likelihood, work risk, institutional-economy risk, interpersonal risk, and psychological risk indexes) could be predicted by various exploratory variables (diagnosis, age, gender, education level, marital status, number of cohabitants, perceived knowledge, control and efficacy, news seeking frequency, perceived knowledge, and affective states). Only significant independent variables are reported. Alpha level set a priori at 0.05. We applied a Bonferroni correction dividing the preset alpha level by the number of variables considered for demographics and perceived risk questionnaire items when comparing the scores by diagnosis and in the correlation matrix, when considering the sample as a whole.

## 3. Results

### 3.1. Socio-Demographic Data

As shown in Table 1, the sample included 56% of males and 44% of females with a median age of 44 years (IQR: 34–53). Most of the participants were single, with higher education and without a stable job. About 70% of patients complied with the collective confinement imposed by the government from March to May 2020, most of the patients were living at home with two to four people (including the patient), and the majority did not have children living with them. The 47.33% of patients had distant family members.

### 3.2. Perception Risk, Risk-Related Variables, and Mortality Risk

As shown in Table 2, after Bonferroni correction, the risk related scores did not differ between the three patients’ groups (*p*-value threshold 0.003). In general, patients were more concerned about the economic and political consequences of COVID-19 than about their own health. Indeed, scores referring to the degree of concern for one’s own life or for the loved ones, as well as score related to the probability of contracting the infection, heal or die from it, settle on a score of 5.67, while the scores related to the economic-political consequences were between 8 and 8.75. In addition, the patients believed that COVID-19 could be solved completely and quickly, and they were strongly concerned about the possible consequences on interpersonal relationships and the psychological impact of COVID-19.

By analyzing the risk-related variables, data showed that patients believed they could control the contagion well, relying on their own self-effectiveness. Results also showed that they considered themselves as adequately informed about the COVID-19 phenomenon, seeking information with a frequency from one to five times a day. Most of them considered the virus created in a lab.

Finally, regarding perceived mortality risk, we found that patients perceived a greater risk of death for cardiovascular or cancer diseases, compared to COVID-19 and infections in general.

### 3.3. Emotional States

As shown in Table 3, after Bonferroni correction, the emotional state scores did not differ between the three patients’ groups (*p*-value threshold 0.003, obtained by dividing the 0.05 by 19 emotions, excluding in the counting the four aggregator factors, to be conservative). In general, patients mainly showed emotions of fear, uncertainty, impotence, nostalgia, loneliness, sadness, and anguish, with higher levels of anxiety, depression, and nervousness. They reported high levels of trust and hope. Combining emotions into groups, positive and anxiety states were more frequent than negative and uncertain ones.

### 3.4. Correlations among Risk Perception and Risk-Related Variables

Spearman’s correlations between different parameters were measured considering the sample as a whole, given the non-statistically significant difference between patients’ groups, as previously described. In particular, the probability of resolution was not associated with any other variables. The health risk perception likelihood of COVID-19 index was associated with a higher perceived risk of mortality for COVID-19 (β = 0.377), infections (β = 0.344), tumor (β = 0.394), and stroke (β = 0.318). Instead, the level of concern for their own health and for the health of their loved ones was related only with COVID-19 (β = 0.420) and infections (β = 0.301). The institutional-economy risk index was linked to the perceived mortality risk due to stroke (β = 0.349), while the interpersonal risk index was related to perceived mortality risk due to heart attack (β = 0.312) and dementia (β = 0.370), and the psychological risk index for heart attack (β = 0.308), stroke (β = 0.390), and tumor (β = 0.329). Finally, negative emotions were correlated to almost all risk-related variables (Work risk index: β = 0.346; Institutional-economy risk index: β = 0.300; Interpersonal risk index: β = 0.322; Health concern index: β = 0.377; Psychological risk index: β = 0.471), while those of uncertainty and anxiety were related only to psychological risk (uncertainty: β = 0.457; anxiety: β = 0.392) and health concern (uncertainty: β = 0.350; anxiety: β = 0.398).

In addition, Spearman’s correlations were performed between all variables of risk perception considering the patients with depression, bipolar disorder, and schizophrenia, separately. These correlations did not differ from the whole sample. The only exception was the health risk perception likelihood, which correlated with anxiety states, and psychological index and Health Concern index in inpatients with Schizophrenia, the health risk perception likelihood with the Institutional index in bipolar patients. The correlation matrices are reported in the Supplementary Tables S1–S3.

### 3.5. The Role of Risk-Related Variables and Risk Perceptions

Table 4 shows the results of multiple univariate regression models, considering the whole sample, but taking into account the patients’ diagnoses as an independent variable. It was found that the likelihood of the complete resolution of COVID-19 index was positively influenced by the diagnosis of schizophrenia. It is predictive to the increased perceived effectiveness and to the presence of positive emotions. Indeed, the presence in the house of more than four people negatively affected this parameter. The concern about health risks index was positively influenced by having distant family members, while the perceived likelihood of contagion and death by the presence of positive emotions. The perception of knowledge of the COVID-19 phenomenon would positively influence both the institutional-economy risk index and the psychological risk index. Finally, positive emotions and uncertainty states would positively influence the interpersonal risk index.

## 4. Discussion

In our study, we evaluated the risk perception of COVID-19 pandemic in a psychiatric population to better understand how patients faced the pandemic in Italy. To our knowledge, no studies have been conducted on psychiatric patients. Between February and March 2020, Diotiaiuti et al. [36] assessed the levels of self-efficacy, risk perception, self-control, and confidence in others and in the government in a sample of university students from central Italy. A significant increase in the risk perception emerged as the government implemented the restrictions, concomitantly resulting in a decrease in self-efficacy and in an increase in policymakers’ decisions. Similarly, Commodari et al. [37] found that self-efficacy and empathy were the elements that most influenced the perception of risk. A study by Ceccato et al. [38] showed that older Italians were moderately more optimistic than younger and middle-aged adults with less fear of contagion. Moreover, older participants reported more negative emotions, and confidence in the news provided by the media. They were also willing to follow restrictive measures and adopt preventive behaviors. On the whole, the aforementioned studies indicate that the general Italian population showed an increase of self-efficacy and confidence in government measure to limit the epidemic.

Our findings showed that there was no difference in the perception of risk among patients with Major Depression, Schizophrenia, and Bipolar Disorder indicating that being affected by a psychiatric illness might influence the risk perception independently from the diagnosis. Indeed, previous studies show that mental illnesses have an impact on individuals’ life across the different condition [39,40], influencing patient’s well-being, daily living, and their healthy habits [23,41,42,43,44,45]. Furthermore, severe psychiatric patients have higher risk of developing COVID-19 probably due to a lack of a sense of self-protection [46].

In our sample, participants reported a higher perceived risk of work and of economic consequences due to COVID-19 pandemic. Data on mortality risk showed that patients considered the risk of dying for heart attack, cancer, and stroke higher than the risk of dying for COVID-19. This is in agreement with the results presented by the group of Lanciano et al. [33], which administered the same questionnaire to the general population of southern Italy during the first phase of the national lockdown. The authors suggest that the Italian population perceived as true risk the limited sense of predictability, controllability, and economic uncertainty due to the pandemic, rather than the virus itself [33]. Furthermore, it is well known that psychiatric patients have greater socio-economic disadvantages than the general population and it is likely that the burden would be enhanced during a national emergency situation [47].

Our results indicate that patients presented a high perception of control, self-efficacy, and positive emotions toward the COVID-19 pandemic, while they were more concerned about the negative impact of the pandemic on psychological well-being and interpersonal relationships. Hence, psychiatric patients were more worried about future consequences of COVID-19 (economic, relational, and mental health) rather than for the immediate danger.

Knowledge about COVID-19 did not affect the risk perception of contagion in the patient’ sample. On the one hand, having more information increased fear for the economic and social consequences adding to the psychological burden. In accordance with the emotion–motivation model [48], we can assume that in the already socially frail psychiatric population [22,49], knowledge about the virus and its transmission dynamics could reduce fear of contagion, while the interplay between affective variables and socio-structural factor might determine a fear of the future [50].

Another point that emerges from our results is that the diagnosis of schizophrenia influenced the perception of COVID-19 resolution. Thus, patients with Schizophrenia appear to have higher levels of hope when facing the COVID-19 threat, imagining a rapid resolution of the emergency and a rapid return to previous life habits. In the literature, it is reported that the levels of hope in inpatients with Schizophrenia do not seem to be affected by the severity of the symptoms assessed with PANSS [51].

The study has several limitations. First, we used an instrument previously validated in a non-clinical population. Thus, our hypotheses need to be further investigated in future studies employing validated instruments in psychiatric patients. The cross-sectional study design prevents us ascertain the causal relationships between variables. In addition, it is to be demonstrated whether the results are generalizable to countries with different heath care systems and social support. We should also acknowledge that the study of temperament traits, previously associated with mental illnesses, perception of stress, and health hazards should be considered in future studies [52,53,54].

## 5. Conclusions

Our results showed that psychiatric patients perceived the economic and social long-term consequences of COVID-19 much more dangerous than the short-term ones (contagion, death, and health risk). In addition, the increase in knowledge about the virus accentuates these concerns. We believe that it is necessary to develop new interventions to implement coping strategies aimed to reduce the impact of emergency situations on the psychiatric population.

## Figures and Tables

**Table 1:** Socio-Demographic Data.

	Total		Major Depression		Bipolar I		Schizophrenia		*p* Value
AGE	44 (IQR: 34–53)		43 (IQR: 33–56)		50 (IQR: 36–59)		42 (IQR: 34–49)		0.086
Gender									
M	84	56.00%	27	54.00%	22	44.00%	35	70.00%	0.030
F	66	44.00%	23	46.00%	28	56.00%	15	30.00%	
Marital Status									
Single	75	50.00%	18	36.00%	22	44.00%	35	70.00%	0.004
Married	39	26.00%	21	42.00%	11	22.00%	7	14.00%	
Separated/Divorced	32	21.33%	9	18.00%	15	30.00%	8	16.00%	
Widowed	4	2.67%	2	4.00%	2	4.00%	0	0.00%	
Education									
Elementary	15	10.00%	7	14.00%	3	6.00%	5	10.00%	0.653
Secondary school	47	31.33%	16	32.00%	15	30.00%	16	32.00%	
High school	70	46.67%	22	44.00%	23	46.00%	25	50.00%	
University and Post-degree	18	12.00%	5	10.00%	9	18.00%	4	8.00%	
Employement									
No	90	60.00%	25	50.00%	31	62.00%	34	68.00%	0.174
Yes	60	40.00%	25	50.00%	19	38.00%	16	32.00%	
Quarantine									
No	9	6.00%	0	0.00%	5	10.00%	4	8.00%	0.006
Yes, I stay home	33	22.00%	18	36.00%	7	14.00%	8	16.00%	
Yes, but I go to work	104	69.33%	28	56.00%	38	76.00%	38	76.00%	
Yes, because I’ve been in contact with a COVID 19 positive	1	0.67%	1	2.00%	0	0.00%	0	0.00%	
Yes, because I tested positive for COVID-19	3	2.00%	3	6.00%	0	0.00%	0	0.00%	
Children at Home									
No	108	72.00%	31	62.00%	39	78.00%	38	76.00%	0.152
Yes	42	28.00%	19	38.00%	11	22.00%	12	24.00%	
Number of Housemates during Quarantine									
Living alone	11	7.33%	3	6.00%	7	14.00%	1	2.00%	0.424
One person	44	29.33%	14	28.00%	15	30.00%	15	30.00%	
Between two and four persons	85	56.67%	29	58.00%	25	50.00%	31	62.00%	
More than four persons	10	6.67%	4	8.00%	3	6.00%	3	6.00%	
CONCERNED ABOUT DISTANT FAMILY MEMBERS	71	47.33%	31	62.00%	23	46.00%	17	34.00%	0.019

**Table 2:** Perception risk, risk-related variables, and mortality risk. IQR: Interquartile range.

			Total		Major Depression		Bipolar I		Schizophrenia		*p* Value
Perception risk and risk-related variables	Likelihood of COVID-19 Resolution		6.5 (IQR: 5–7.5)		6.00 (IQR: 5–7)		6.5 (IQR: 5.5–8)		7 (IQR: 5.5–8)		0.011
	Health Concern		5.66 (IQR: 4–7)		5.33 (IQR: 4.33–7)		5.67 (IQR: 3.67–7)		6 (IQR: 3.33–7.33)		0.946
	Health Likelihood (contagion, death, healing reversed)		5.67 (IQR: 5–6.5)		5.83 (IQR: 5–6.5)		5.5 (IQR: 5–6.5)		5.67 (IQR: 4.67–6.83)		0.91
	Work Risk		8 (IQR: 7.2–9)		8 (IQR: 7.4–8.8)		8 (IQR: 7.2–9)		8 (IQR: 6.8–9.6)		0.946
	Institutional-Economic Risk		8.75 (IQR: 7.5–9.5)		9 (IQR: 7.75–9.25)		8.25 (IQR: 7.5–9.75)		8.5 (IQR: 7–10)		0.773
	Interpesonal Risk		7 (IQR: 5.25–8.25)		6.25 (IQR: 5.5–8)		7 (IQR: 5–8.25)		7.25 (IQR: 5.25–9)		0.558
	Psychological Risk		7.6 (IQR: 6.20–9)		8 (IQR: 6.80–9.40)		7.6 (IQR: 6–9)		7.2 (IQR: 6–8)		0.212
	Perceived Knowledge		7 (IQR: 6–8)		8 (IQR: 7–8)		7 (IQR: 6–8)		7 (IQR: 6–8)		0.066
	COVID-19 Cause	Bat	13	−8.70%	1	−2%	4	−8%	8	−16%	
		Virus created in laboratory	36	−24%	13	−26%	10	−20%	13	−26%	
		Chemical/Economic/Social War	27	−18%	8	−16%	9	−18%	10	−20%	
		Evolution of an existing virus/Species jump	24	−16%	11	−22%	8	−16%	5	−10%	
		I don’t know	20	−13.30%	8	−16%	7	−14%	5	−10%	0.536
		Experiment disclosed by mistake	19	−12.70%	7	−14%	7	−14%	5	−10%	
		Chinese eating dogs	3	−2%	1	−2%	2	−4%	0	0%	
		Divine providence	4	−2.70%	0	0%	2	−4%	2	−4%	
		Negligence	2	−1.30%	0	0%	1	−2%	1	−2%	
		Climate change	1	−0.70%	1	−2%	0	0%	0	0%	
		Reduction of world population	1	−0.70%	0	0%	0	0%	1	−2%	
	News Seeking/Day	0	21	−14.10%	7	−14%	9	−18%	5	−10%	
		From 1 to 5	109	−72.70%	38	−76%	37	−74%	34	−68%	0.31
		From 5 to 10	16	−10.70%	4	−8%	3	−6%	9	−18%	
		>10	4	−2.70%	1	−2%	1	−2%	2	−4%	
	Perceived Efficacy		7 (IQR: 5.75–7.75)		7.25 (IQR: 5.5–7.75)		6.75 (IQR: 6–7.75)		7 (IQR: 5.75–7.75)		0.912
	Perceived Control		7 (IQR: 5–8)		6 (IQR: 5–8)		7 (IQR: 5–8)		7 (IQR: 5–8)		0.183
Mortality Risk	COVID-19		6 (IQR: 5–8)		7 (IQR: 5–7)		6 (IQR: 4–8)		7 (IQR: 5–9)		0.28
	Heart Attack		8 (IQR: 6–9)		8 (IQR: 7–8)		7 (IQR: 6–8)		7 (IQR: 5–9)		0.388
	Stroke		7 (IQR: 6–8)		8 (IQR: 7–8)		7 (IQR: 6–8)		7 (IQR: 5–8)		0.202
	Cancer		8 (IQR: 6–9)		8 (IQR: 7–9)		7 (IQR: 6–9)		8 (IQR: 6–10)		0.266
	Dementia		6 (IQR: 5–7)		6 (IQR: 5–7)		6 (IQR: 4–7)		6 (IQR: 5–8)		0.071
	Infection		6 (IQR: 5–7)		6 (IQR: 5–7)		6 (IQR: 5–7)		6 (IQR: 5–8)		0.657

**Table 3:** Emotional states.

	Total	Major Depression	Bipolar I	Schizophrenia	*p* Value
Anger	6 (IQR: 3–8)	7 (IQR: 3–9)	7 (IQR: 3–9)	5 (IQR: 2–7)	0.025
Wrath	4 (IQR: 1–7)	6 (IQR: 2–8)	4 (IQR: 2–8)	3 (IQR: 1–6)	0.452
Fear	7 (IQR: 5–8)	8 (IQR: 5–10)	7 (IQR: 5–8)	7 (IQR: 4–8)	0.205
Trust	7 (IQR: 6–9)	7 (IQR: 6–9)	8 (IQR: 6–9)	7 (IQR: 6–10)	0.828
Hope	8 (IQR: 6–9)	8 (IQR: 7–9)	8 (IQR: 6–9)	7 (IQR: 6–10)	0.629
Vulnerability	6 (IQR: 4–8)	6 (IQR: 4–8)	7 (IQR: 5–8)	6 (IQR: 2–7)	0.142
Injustice	6 (IQR: 3–8)	6 (IQR: 2–8)	6 (IQR: 3–9)	5 (IQR: 4–8)	0.616
Frustration	5 (IQR: 3–8)	6 (IQR: 4–8)	6 (IQR: 2–8)	5 (IQR: 1–7)	0.029
Disorientation	5 (IQR: 3–8)	6 (IQR: 4–8)	7 (IQR: 2–8)	5 (IQR: 2–7)	0.154
Confusion	6 (IQR: 4–8)	7 (IQR: 5–8)	7 (IQR: 5–8)	5 (IQR: 4–7)	0.013
Uncertainty	7 (IQR: 5–8)	7 (IQR: 6–9)	7 (IQR: 4–9)	6 (IQR: 5–8)	0.126
Inadequacy	6 (IQR: 3–8)	6 (IQR: 2–7)	6 (IQR: 2–8)	5 (IQR: 3–7)	0.303
Impotence	7 (IQR: 4–9)	8 (IQR: 6–10)	7 (IQR: 2–7)	6 (IQR: 4–8)	0.036
Nostalgia	7 (IQR: 5–9)	8 (IQR: 5–10)	7 (IQR: 3–9)	7 (IQR: 5–8)	0.191
Disquiet	6 (IQR: 4–8)	6 (IQR: 5–8)	6 (IQR: 4–8)	6 (IQR: 4–7)	0.736
Solitude	7 (IQR: 4–9)	8 (IQR: 5–10)	7 (IQR: 4–9)	7 (IQR: 5–8)	0.348
Anxiety	7 (IQR: 4–8)	7 (IQR: 5–10)	7 (IQR: 4–8)	6 (IQR: 3–8)	0.028
Nervousness	7 (IQR: 4–8)	7 (IQR: 5–9)	7 (IQR: 5–8)	6 (IQR: 4–7)	0.012
Depression	7 (IQR: 4–9)	8 (IQR: 4–10)	8 (IQR: 3–10)	5 (IQR: 3–7)	0.009
Sadness	7 (IQR: 5–8)	8 (IQR: 6–8)	7 (IQR: 4–9)	7 (IQR: 5–8)	0.109
Anguish	7 (IQR: 4–8)	7 (IQR: 6–9)	7 (IQR: 4–8)	5 (IQR: 4–7)	0.013
Negative Affective States index	5.94 (IQR: 4.5–7.63)	6.63 (IQR: 4.75–7.5)	6.13 (IQR: 4.10–8)	5.69 (IQR: 4.5–6.78)	0.043
Positive Affective States index	7.5 (IQR: 4.5–7.63)	7.5 (IQR: 6–9)	8 (IQR: 6–9)	7 (IQR: 6–9.25)	0.058
Uncertain States index	5.8 (IQR: 4.5–7.63)	6.17 (IQR: 4.5–7.88)	6.17 (IQR: 4.88–8.04)	5.3 (IQR: 3.78–6.41)	0.054
Anxiety States index	6.25 (IQR: 4.75–7.5)	6.5 (IQR: 5–7.56)	6.25 (IQR: 4.18–7.75)	5.5 (IQR: 4.38–7)	0.909

**Table 4:** Multiple regression analyses of the perceived risk indices *.

	Likelihood of COVID-19 Resolution Index		Health Concern Index		Health Likelihood		Work Risk Index		Institutional-Economy Risk Index		Interpersonal Risk Index		Psychological Risk Index	
	β	t	β	t	β	t	β	t	β	t	β	t	β	t
(Constant)		2.47				2.96		3.82		4.41				3.01
Diagnosis (Reference depression)														
Bipolar disorder														
Schizophrenia	0.27	2.56												
Cohabitants (reference alone)														
One person														
Two-four persons														
More than four persons	−0.3	−2.53												
Concern about distant family members			0.21	2.64										
Perceived knowledge									0.2	2.09			0.21	2.15
Uncertainty states											0.27	2.11		
Positive states	0.24	2.7			0.2	2.19					0.22	2.59		

* Dependent variables measured: diagnosis, age, gender, education, occupation, civil state, cohabitants, concern about distant family members, perceived knowledge, COVID-19 news seeking, perceived control, perceived efficacy, negative states, anxiety states, uncertainty states, and positive states. Only significant (*p* < 0.050) independent variables are reported.

## Data Availability

The data presented in this study are available on request from the corresponding author.

## Supplementary Materials

The following supporting information can be downloaded at: https://www.mdpi.com/article/10.3390/ijerph19052620/s1, Table S1. Correlation matrix of COVID-19 risk related variables for patients with depression; Table S2. Correlation matrix of COVID-19 risk related variables for patients with bipolar disorder A; Table S3. Correlation matrix of COVID-19 risk related variables for patients with schizophrenia dd the titles.
