# Supplementary material for: Risk Perception among Psychiatric Patients during the COVID-19 Pandemic
Source: Int J Environ Res Public Health. 2022 Feb 24;19(5):2620. doi: 10.3390/ijerph19052620 (PMC8909657; doi:10.3390/ijerph19052620)
Supplement: Supplementary file 1 [file ijerph-19-02620-s001.zip › ijerph-1511969-supplementary.pdf]

Table S1. Correlation matrix of COVID-19 risk related variables for patients with depression

	Mortality INFARTO	Perceived mortality risk for STROKE	Negative states	Anxiety states
Perceived mortality risk for STROKE	**,871***	--		
Perceived mortality risk for CANCER	**,673***	**,567***		
Anxiety states	,116	,133	**,666***	--
Uncertainty states	,440	,368	**,633***	**,594***
Psychological risk index	**,596***	**,519***	,364	,222

The Spearman correlation matrix considered the following variables: Perceived mortality risk for COVID 19, heart attack, stroke, cancer, dementia or infection, perceived control, perceived knowledge, negative states, anxiety states, uncertainty states, positive states, Likelihood of COVID-19 resolution index, health likelihood index, work risk index, Institutional-economy risk index, interpersonal risk index, health concern index, psychological risk index, efficacy index. The numbers in **bold** with the asterisk (*) indicate the correlations that stay significant after Bonferroni correction (p-value < 0.00025). Non-significant correlations not shown.

Table S2. Correlation matrix of COVID-19 risk related variables for patients with bipolar disorder

	Perceived mortality risk for COVID 19	Perceived mortality risk for HEART ATTACK	Perceived mortality risk for STROKE	Perceived mortality risk for CANCER	Perceived mortality risk for DEMENTIA	Negative states	Anxiety states	Uncertainty states	Health likelihood index	Work risk index
Perceived mortality risk for STROKE	,447	**,747***	--							
Perceived mortality risk for CANCER	,381	**,531***	**,514***	--						
Perceived mortality risk for DEMENTIA	**,516***	**,544***	**,560***	**,654***	--					
Perceived mortality risk for INFECTION	**,571***	,407	,384	**,549***	,447					
Anxiety states	,275	,202	,280	,216	,272	**,789***	--			
Uncertainty states	,249	,228	,446	,170	,196	**,666***	**,775***	--		
Institutional-economy risk index	,327	,269	,323	,357	,246	**,576***	,424	,378	**,502***	**,659***
Interpesonal risk index	,385	,440	,411	,430	**,616***	**,522***	,319	,299	,190	,464
Psychological risk index	,214	,288	,364	,239	,209	,493	,429	,516	,178	**,569***

The Spearman correlation matrix considered the following variables: Perceived mortality risk for COVID 19, heart attack, stroke, cancer, dementia or infection, perceived control, perceived knowledge, negative states, anxiety states, uncertainty states, positive states, Likelihood of COVID-19 resolution index, health likelihood index, work risk index, Institutional-economy risk index, interpersonal risk index, health concern index, psychological risk index, efficacy index. The numbers in **bold** with the asterisk (*) indicate the correlations that stay significant after Bonferroni correction (p-value < 0.00025). Non-significant correlations not shown.

Table S3. Correlation matrix of COVID-19 risk related variables for patients with schizophrenia

	Perceived mortality risk for HEART ATTACK	Perceived mortality risk for STROKE	Perceived mortality risk for CANCER	Perceived mortality risk for CANCER	Negative states	Anxiety index	Health likelihood index	Work risk index	Interpersonal risk index	Health concern index
Perceived mortality risk for STROKE	**,693***	--								
Perceived mortality risk for CANCER	,404	**,573***	--							
Perceived mortality risk for DEMENTIA	,102	,232	**,616***	--						
Perceived mortality risk for INFECTION	,284	,371	,441	**,543***						
Anxiety states	,043	,107	,148	,264	**,827***	--				
Uncertainty states	-,055	-,135	-,099	,270	**,609***	**,643***				
Health likelihood index	,286	,347	,470	,298	,302	**,509***	--			
Interpesonal risk index	,217	,130	,193	,192	,227	,176	,425	**,682***	--	
Health concern index	-,010	,009	,287	,309	,378	**,546***	,485	,109	,170	--
Psychological risk index	,045	,231	,304	,331	,496	**,550***	**,515***	,292	**,537***	**,501***

The Spearman correlation matrix considered the following variables: Perceived mortality risk for COVID 19, heart attack, stroke, cancer, dementia or infection, perceived control, perceived knowledge, negative states, anxiety states, uncertainty states, positive states, Likelihood of COVID-19 resolution index, health likelihood index, work risk index, Institutional-economy risk index, interpersonal risk index, health concern index, psychological risk index, efficacy index. The numbers in **bold** with the asterisk (*) indicate the correlations that stay significant after Bonferroni correction (p-value < 0.00025). Non-significant correlations not shown.
